# Are posttraumatic stress disorder (PTSD) and complex-PTSD distinguishable within a treatment-seeking sample of Syrian refugees living in Lebanon?

**DOI:** 10.1017/gmh.2018.2

**Published:** 2018-04-16

**Authors:** P. Hyland, R. Ceannt, F. Daccache, R. Abou Daher, J. Sleiman, B. Gilmore, S. Byrne, M. Shevlin, J. Murphy, F. Vallières

**Affiliations:** 1 Centre for Global Health, University of Dublin, Trinity College, 7-9 South Leinster Street, Dublin 2, Ireland; 2 School of Business, National College of Ireland, International Financial Services Centre, Mayor Street, Dublin 1, Ireland; 3 International Medical Corps Lebanon, Beirut, Lebanon; 4 School of Psychology, University of Dublin, Trinity College, Ireland; 5 Psychology Research Institute, Ulster University, Londonderry, Northern Ireland

**Keywords:** Complex posttraumatic stress disorder (CPTSD), ICD-11, refugees, posttraumatic stress disorder (PTSD)

## Abstract

**Background:**

The World Health Organization will publish its 11^th^ revision of the International Classification of Diseases (ICD-11) in 2018. The ICD-11 will include a refined model of posttraumatic stress disorder (PTSD) and a new diagnosis of complex PTSD (CPTSD). Whereas emerging data supports the validity of these proposals, the discriminant validity of PTSD and CPTSD have yet to be tested amongst a sample of refugees.

**Methods:**

Treatment-seeking Syrian refugees (*N* = 110) living in Lebanon completed an Arabic version of the *International Trauma Questionnaire*; a measure specifically designed to capture the symptom content of ICD-11 PTSD and CPTSD.

**Results:**

In total, 62.6% of the sample met the diagnostic criteria for PTSD or CPTSD. More refugees met the criteria for CPTSD (36.1%) than PTSD (25.2%) and no gender differences were observed. Latent class analysis results identified three distinct groups: (1) a PTSD class, (2) a CPTSD class and (3) a low symptom class. Class membership was significantly predicted by levels of functional impairment.

**Conclusion:**

Support for the discriminant validity of ICD-11 PTSD and CPTSD was observed for the first time within a sample of refugees. In support of the cross-cultural validity of the ICD-11 proposals, the prevalence of PTSD and CPTSD were similar to those observed in culturally distinct contexts.

The World Health Organization's (WHO) *11^th^ version of the International Classification of Diseases* (ICD-11) will be published in 2018. The ICD-11 puts forward substantial revisions, guided by key organising principles regarding cross-cultural validity and clinical utility (see Maercker *et al.*, 2013). Notably, the ICD-11 model of post-traumatic stress disorder (PTSD) includes only six symptoms; markedly fewer than the 20 symptom model of PTSD outlined within the 5^th^ edition of the *Diagnostic and Statistical Manual of Mental Disorders* (American Psychiatric Association [APA], 2013). These six symptoms are grouped into three clusters: (i) Re-experiencing of the trauma in the here and now (Re); (ii) deliberate avoidance of traumatic reminders (Av); and (iii) a sense of current threat (Th) (Maercker *et al.*, 2013), with two symptoms represented under each cluster. To date, several studies have provided empirical support for the proposed factor structure of PTSD within diverse trauma samples (e.g. Hansen et al., 2015; Hyland *et al*., 2017a; La Greca *et al*., 2017).

The ICD-11 will also introduce complex-PTSD (CPTSD) into the diagnostic nomenclature for the first time (Maercker *et al.*, 2013). CPTSD includes the aforementioned six core PTSD symptoms and an additional three clusters of symptoms. Collectively referred to as ‘Disturbances in Self-Organisation’ (DSO), these additional three clusters are intended to capture the more pervasive psychological disturbances that can arise following traumatic exposure and include: (i) affective dysregulation (AD), reflecting both hyperactivation and hypoactivation of emotional responses; (ii) Negative Self-Concept (NSC), reflecting extreme negative self-evaluations; and (iii) disturbed relationships (DR), reflecting a tendency to avoid interpersonal relationships. Several factor analytic studies have provided support for the proposed factorial validity of CPTSD (e.g. Hyland *et al.*, 2017b, c), including one study of refugees (Nickerson *et al.*, 2016).

While traumatic exposure is a diagnostic requirement for PTSD and CPTSD, it is considered a risk-factor, rather than a determining factor, for a differential diagnosis of PTSD or CPTSD (Cloitre *et al.*, 2013). Specifically, exposure to multiple forms of trauma, particularly early-life interpersonal trauma, has been shown to elevate the risk of CPTSD (Hyland *et al.*, 2017b), whereas single-incident traumatic exposure, particularly when occurring later in development, has been shown to elevate risk of PTSD (Cloitre *et al.*, 2013). This conceptualisation of traumatic exposure as a risk-factor (rather than a requirement) for a differential diagnosis allows for the occurrence of CPTSD within a particularly vulnerable individual following a single-incident trauma, and the occurrence of PTSD (or no diagnosis) within a resilient individual following exposure to multiple and/or early-life traumas. In addition to trauma history, several demographic and psychosocial factors have also been identified as risk factors for CPTSD. Individuals with CPTSD are more likely to be unemployed, unmarried and living alone (Hyland *et al.*, 2017c; Karatzias *et al.*, 2017). Additionally, females are twice as likely as males to be diagnosed with PTSD and CPTSD (Karatzias *et al.*, 2017). Individuals diagnosed with CPTSD are also repeatedly found to experience significantly higher levels of psychosocial impairment (Elklit *et al*., 2014; Murphy *et al*., 2016).

Admittedly, the effort to identify factors that differentially predict CPTSD and PTSD assumes that these two disorders are, in fact, distinct. Some authors have argued that no such distinction exists, and that CPTSD is essentially indistinguishable from the broad-based description of PTSD provided by the DSM-5 (Wolf *et al.*, 2015). Empirical assessments of the discriminant validity of ICD-11 PTSD and CPTSD have generally relied on the use of a statistical technique called latent class analysis (LCA) (or latent profile analysis (LPA)). LCA/LPA are referred to as ‘person-centred’ statistics and allow for the identification of homogeneous ‘classes’ of individuals through the examination of patterns of responses to categorical (LCA) or continuous (LPA) data (Debowska *et al*., 2017). Both approaches assume that observed response patterns (e.g. PTSD and DSO symptom endorsements) can be explained by a finite set of mutually exclusive latent classes. To date, there have been ten LCA/LPA-based studies conducted across different countries (e.g. USA, Germany, Denmark, UK and Uganda), and with samples exposed to varying forms of traumatic exposure (e.g. institutional child abuse, sexual assault, bereavement, child soldiering). Of these ten studies, eight offer support for qualitatively distinct classes where symptom profiles are consistent with the distinction between ICD-11 PTSD and CPTSD (Cloitre *et al.*, 2013, 2014; Elklit *et al.*, 2014; Knefel *et al.*, 2015; Perkonigg *et al.*, 2015; Murphy *et al.*, 2016; Karatzias *et al.*, 2017; Sachser *et al.*, 2017). In contrast, the remaining two studies (Wolf *et al*., 2015; Gluck *et al.*, 2016) observed classes that differed quantitatively rather than qualitatively. These studies, therefore, suggest that CPTSD is not distinguishable from PTSD and that differences in classes are best explained varying degrees of symptom severity on a single, underlying, condition (i.e. PTSD).

Taken together, the existing LCA/LPA literature seems to offer stronger support for two distinct trauma-based disorders, as put forward in the ICD-11. However, this body of research is not without limitations. To date, only one study has been performed amongst a non-Western cultural sample (Murphy *et al.*, 2016) and no studies have thus far evaluated the discriminant validity of PTSD and CPTSD amongst a sample of refugees. Given the WHO's guiding principles of clinical utility, as well as their emphasis on cross-cultural validity for the ICD-11, more research is needed to ensure the validity of ICD-11 PTSD and CPTSD in low-resource, humanitarian settings (Brewin, 2013) and amongst refugee populations. Furthermore, only two studies (Murphy *et al.*, 2016; Karatzias *et al.*, 2017) have assessed the discriminant validity of PTSD and CPTSD using the *International Trauma Questionnaire* (ITQ: Cloitre *et al.*, 2015), a self-report measure specifically designed to capture the ICD-11 PTSD and CPTSD symptoms. While both of these studies support the distinguishability of these diagnoses, further studies are required to support the use of this scale for the assessment of these diagnoses.

Therefore, and with a view to addressing these limitations, the current study sought to evaluate the discriminant validity of ICD-11 PTSD and CPTSD amongst a treatment-seeking sample of Syrian refugees living in Lebanon, using an Arabic translation of the ITQ. This goal was achieved through three specific research objectives. First, we sought to identify the prevalence of ICD-11 PTSD and CPTSD. Second, we sought to determine whether there were any emerging unique latent classes of refugees, and if so, whether these symptom profiles are consistent with the distinct diagnoses of the ICD-11. Finally, we explored whether there were any relationships between the observed classes and a range of socio-demographic and trauma-related variables.

## Methods

### Participants and procedures

Participants were 110 treatment-seeking Syrian refugees living in Lebanon (80.2% female, mean age = 33.02, s.d. = 8.94). The majority were unemployed (75.5%, *n* = 80), with a mean of 5.71 years of education (s.d. = 4.39, range 0–18 years). Most participants were registered with the office of United Nations High Commissioner for Refugees (90.1%, *n* = 100) and had spent an average of 37.45 months (s.d. = 14.62) in Lebanon. A small proportion of the sample resided within a refugee camp (12.7%, *n* = 14), with the majority living with family members or friends (96.4%, *n* = 107). In line with traumatic exposure being a diagnostic requirement for PTSD and CPTSD, all participants indicated exposure to a traumatic life event. The traumatic events reported as most distressing were forced displacement (18.5%, *n* = 20), exposure to bomb blasts (10.2%, *n*  = 11), the sudden and unexpected death of a loved one (10.2%, *n* = 11) and exposure to warfare (7.4%, *n* = 8). A small proportion of individuals reported a childhood traumatic (e.g. sexual or physical abuse) event as their most distressing trauma (2.7%, *n* = 3).

Participants were recruited through International Medical Corps’ (IMC) Lebanon Mental Health Program. IMC case managers identified participants, informed them of the study and gave them the option of participation, before referring them to one of eight IMC psychotherapists. Case managers were affiliated with a total of 11 primary health care centers across four (Beqaa, Beirut/Mount Lebanon, North Lebanon and South Lebanon) of eight provinces (*muhafazah*). To participate, individuals had to be over the age of 18 and forcibly displaced to Lebanon from Syria within the last five years. Given the low rates of literacy, psychotherapists were instructed to administer the scale to those who had provided consent. All psychotherapists were trained to administer the ITQ during a one-day workshop held in Beirut in November 2015, during which case managers were also trained on how to obtain informed consent. Ethical approval was obtained from the Health Policy & Management/Centre for Global Health Research Ethics Committee, Trinity College Dublin and from the Comité d’Éthique, Université Saint Joseph, Beirut, Lebanon. Written or verbal consent was obtained, depending on the literacy of the participant. In the case of an illiterate participant, verbal consent was obtained and signatures were given in the form of a thumbprint.

### Measures

An Arabic translation of the International Trauma Questionnaire (ITQ Version 1.3: Cloitre *et al.*, 2015) was used to measure symptoms of PTSD and DSO. The ITQ was professionally translated into Arabic and back-translated into English to ensure consistency and that nothing was lost in translation. The ITQ first asks the respondent to report their most distressing traumatic event, and how long ago the event occurred.

To measure PTSD symptoms, respondents are instructed to indicate how often in the past month they have experienced each of the six symptoms (Re1–Re2, Av1–Av2, Th1–Th2). In addition, three items measure functional impairment (impairment in social, work and family/other important areas of life). Diagnosis requires the presence at least one symptom from the Re, Av and Th clusters, plus endorsement of one indicator of functional impairment. To measure the ‘Disturbances in Self-Organisation’ (DSO) symptoms, respondents are instructed to indicate how they typically feel, think about themselves and relate to others. There are a total of 16 DSO symptom indicators. The AD factor is measured using nine items, five of which measure ‘AD-hyperactivation’ (AD1-AD5) and four of which measure ‘AD-hypoactivation’ (AD6-AD9). Four items are used to measure negative self-concepts (NSC1–NCS4); and three items are used to Disturbed Relationships (DR1–DR3).

Individuals responded to all PTSD and DSO items using a five-point Likert response format, ranging from ‘Not at all’ (0) to ‘Extremely’ (4). For diagnostic purposes, and consistent with recommendations in the trauma literature (Elklit & Shevlin, 2007), a symptom (and an indicator of functional impairment) was considered to be present based on a response of ⩾2 (‘Moderately’). Diagnosis of CPTSD requires that the PTSD criteria are met plus the following scores for each of the DSO clusters: (i) A score of ⩾10 for AD-hypoactivation *or* a score of ⩾8 for AD-hypoactivation; (ii) a score ⩾8 for NSC; and (iii) a score ⩾6 for DR.

Although the ITQ is still a preliminary-stage measure of ICD-11 PTSD and CPTSD symptoms, and is intended to undergo further revisions so as to align with the finalised ICD-11 guidelines when officially published in 2018, initial evidence supports the psychometric properties of the measure (e.g. Karatzias *et al*., 2016; Hyland *et al.*, 2017c). The internal reliability of the PTSD (Cronbach's *α* = 0.76) and DSO (Cronbach's *α* = 0.88) subscales were satisfactory in the current sample. A summed total score of functional impairment was also calculated, whereby higher scores reflect higher levels of impairment. The functional impairment items demonstrated good internal reliability (Cronbach's *α* = 0.82).

### Analysis

The analytical plan for the current study included three steps, where each step corresponded to one of the three study objectives. First, prevalence estimates of ICD-11 PTSD and CPTSD were calculated along with assessments of gender differences using a chi-square analysis. Second, an LCA was performed based on the probability of meeting the diagnostic criteria for the three PTSD (Re, Av, Th) and four DSO (AD-hyperactivation, AD-hypoactivation, NSC, DR) symptom clusters. Six latent class models were tested (1–6 classes) using the robust maximum-likelihood estimator (Yuan & Bentler, 2000), and all models were estimated using full information. To avoid solutions based on local maxima, 500 random sets of starting values were used, followed by 50 final stage optimisations. The relative fit of the models was compared using three information theory based fit statistics: The Akaike Information Criterion (AIC; Akaike, 1987), the Bayesian Information Criterion (BIC; Schwartz, 1978) and the sample size-adjusted Bayesian Information Criterion (ssaBIC; Sclove, 1987). The class solution with the lowest value on each fit statistic can be judged to be the best model. The bootstrap likelihood ratio test (BLRT) was also used to compare models with increasing numbers of latent classes. A non-significant value (*p* > 0.05) for the BLRT indicates that the model *with one less class* should be accepted. The BLRT test used 500 bootstrap samples and 50 random start values followed by 20 final stage optimisations. Evidence from simulation studies indicates that the BIC is the best information criterion for identifying the correct number of classes, and the BLRT is the optimal test to select the correct class solution (Nylund *et al.*, 2007). The determination of the best class solution was therefore focused on these two statistics. These analyses were conducted using Mplus version 7.11 (Muthén & Muthén, 2013).

Finally, the relationship between class membership and nine covariates were assessed by means of logistic regression. The covariates in the model included functional impairment, age, number of years spent in education, the number of months spent in Lebanon as a refugee, gender (0 = male, 1 = female), marital status (0 = married or single, 1 = separated, divorced, or widowed), employment status (0 = employed, 1 = unemployed), living status (0 = living with family and/or friends, 1 = living in a refugee camp) and identification of a trauma directly related to one's refugee status (e.g. displacement, bomb blasts, warfare, etc.) as most distressing (0 = no, 1 = yes).

## Results

### Diagnostic rates

The proportion of refugees meeting diagnostic criteria for each PTSD and DSO cluster are reported in Table 1. Positive diagnostic status was high for each PTSD cluster and slightly lower for the DSO clusters. In total, 62.6% (*n* = 67) of refugees met the criteria for a diagnosis of PTSD or CPTSD. As the ICD-11 taxonomic structure only permits one diagnosis (PTSD or CPTSD, not both), disorder-specific diagnostic rates were calculated. A slightly greater number of people met diagnostic status for CPTSD (36.1%, *n* = 39) than PTSD (25.2%, *n* = 27). There were no significant gender differences in the diagnostic rates for PTSD (χ^2^ = 0.60, df = 1, *p* = 0.437, OR =  0.63) or CPTSD (χ^2^ = 0.31, df = 1, *p* = 0.578, OR = 1.31).

**Table 1.** Frequencies and percentages of refugees meeting diagnostic criteria for each PTSD and DSO symptom cluster, and PTSD and CPTSD diagnosis (N = 110)

	*N*	%
Re-experiencing	87	79.1
Avoidance	93	84.5
Sense of threat	95	86.4
Affective dysregulation – hyperactivation	78	71.6
Affective dysregulation – dypoactivation	75	68.2
Affective dysregulation	91	82.7
Negative self-concept	65	59.6
Disturbed relationships	70	63.6
PTSD or CPTSD	67	62.6
PTSD	27	25.2
CPTSD	39	36.1

### LCA results

The LCA results (Table 2) were somewhat equivocal in that the BIC favoured a two-class solution and the BLRT favoured a three-class solution. The three-class solution was selected for two reasons. First, Nylund *et al.*’s (2007) simulation analysis demonstrated that the BLRT was the best statistic by which to select the optimal class solution; and second, inspection of the profile plot for the three-class solution provided a more theoretically interpretable set results.

**Table 2.** Fit indices for the LCA (N = 110)

Classes	Log likelihood	AIC	BIC	ssaBIC	BLRT (*p*)
1	−427.077	868.154	887.058	864.937	−
2	−360.161	750.322	790.830	743.429	133.832 (<0.001)
**3**	**−347.654**	**741.308**	**803.419**	**730.738**	**25.015 (<0.001)**
4	−340.543	743.086	826.801	728.839	14.222 (0.162)
5	−334.550	747.099	852.418	729.177	11.986 (0.500)
6	−332.584	759.169	886.091	737.570	3.931 (1.00)

Note: AIC, Akaike Information Criterion; BIC, Bayesian Information Criterion; ssaBIC, sample size-adjusted BIC; BLRT , Bootstrap likelihood ratio test; Best-fitting model in bold.

**Fig. 1.**
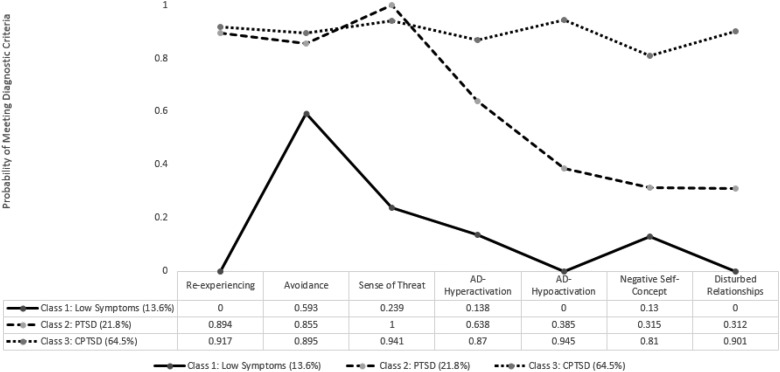
Profile plot based on the best-fitting three-class solution from the LCA.

The profile plot for the three-class solution is displayed in Fig. 1. Class 1 (13.6%, *n* = 15) was the smallest class and was characterised by low probabilities of meeting the diagnostic criteria for each of the PTSD and DSO symptom clusters. This class was labelled the ‘low symptom’ class. Class 2 (21.8%, *n* = 24) was characterised by high probabilities of meeting the diagnostic criteria for each of the PTSD symptom cluster (Re, Av and Th) and low probabilities of meeting the diagnostic criteria for each of the DSO symptom clusters (AD-Hyperactivation, AD-hypoactivation, NSC and DR). This class was labelled the ‘PTSD’ class. Class 3 (64.5%, *n* = 71) was the largest class and was characterised by high probabilities of meeting the diagnostic criteria for each of the PTSD and DSO symptom clusters. This class was labelled the ‘CPTSD’ class.

### Correlates of class membership

A multinomial logistic regression analysis was performed to determine the relationship between each of the demographic and trauma-related variables and one's class membership (see Table 3 for full results). The ‘low symptom class’ was treated as the reference category for these analyses. The model as a whole was statistically significant (χ^2^ = 46.15, df = 18, *p* < 0.001) and explained 46.8% of variance in class membership (Nagelkerke = 0.468). Of the nine predictors in the model only functional impairment was significantly associated with class membership. Increased levels of functional impairment were associated with an increased likelihood of PTSD class membership (OR = 1.38, *p* = 0.032) and CPTSD class membership (OR = 1.81, *p* < 0.001). Although not reaching the level of statistical significance, gender, unemployment status and traumatic history were robustly and positively associated with PTSD and CPTSD class membership.

**Table 3.** Multinomial logistic regression results predicting PTSD and CPTSD class membership (N = 110)

Predictor variables	PTSD class	CPTSD class
	OR (95% CI)	OR (95% CI)
Years of education	0.96 (0.76–1.20)	0.89 (0.71–1.11)
Age	0.94 (0.85–1.04)	0.97 (0.88–1.06)
Functional impairment	1.38 (1.03–1.85)*	1.81 (1.34–2.43)**
Months spent in Lebanon	1.00 (0.95–1.06)	1.02 (0.96–1.08)
Gender	2.35 (0.24–23.38)	2.83 (0.30–26.85)
Marital status	1.33 (0.11–15.49)	0.51 (0.05–5.52)
Employment status	4.96 (0.58–42.33)	4.27 (0.50–36.45)
Living status	0.60 (0.06–6.35)	1.06 (0.09–11.99)
Trauma type	2.13 (0.31–14.76)	6.01 (0.80–45.08)

Note: Reference group for all analyses is the ‘low symptom class’ from the LCA analysis; *p < 0.05, **p < 0.001.

## Discussion

Results revealed a high level of PTSD (25.2%) and CPTSD (36.1%) amongst the current sample of treatment-seeking Syrian refugees. The combined PTSD/CPTSD prevalence rate in this sample of refugees was substantially higher than the 30% prevalence rate of PTSD (as per the DSM guidelines) reported in a previous meta-analytic study of traumatised refugees (Steel *et al.*, 2009). This increased incidence of PTSD/CPTSD amongst the current sample is likely attributable to the treatment-seeking nature of these individuals. Current prevalence rates are, however, comparable with those reported by Nickerson *et al.* (2016) who studied an internationally diverse, treatment-seeking sample of refugees resettled in Switzerland (PTSD = 19.7%, CPTSD = 32.8%). Moreover, current estimates of PTSD/CPTSD prevalence align with figures from treatment-seeking adults in Denmark (49.1%: Hyland *et al.*, 2017*b*) and the UK (64.5%: Hyland *et al.*, 2017*c*). The similarity in prevalence rates of PTSD/CPTSD amongst culturally diverse clinical samples, including among Syrian refugees in Lebanon, offers tentative support for the international applicability and cross-cultural validity of the ICD-11 diagnoses of PTSD and CPTSD. While additional research with clinical and community samples from across the world is necessary before any firm statements regarding cross-cultural validity can be made, the initial data is promising.

There were no significant gender differences in the diagnostic rates of PTSD and CPTSD, a finding that stands in contradiction with the majority of the existing trauma literature (Christiansen & Hansen, 2014). Interestingly, a recent study among former child soldiers in Uganda also found no gender differences in risk for PTSD and CPTSD diagnosis (Murphy *et al.*, 2016). As rates of PTSD and CPTSD were extremely high among both samples, it is possible that any gender variation was nullified at these extreme levels of distress. Alternatively, it may be the case that regularly observed gender differences in PTSD/CPTSD amongst Western populations are not as prevalent in culturally distinct contexts.

The LCA findings were generally supportive of the discriminant validity of PTSD and CPTSD. The minor inconsistency between the BIC and BLRT statistics suggests that these results should be interpreted with caution, and may be the result of a limited sample size. However, the three-class solution, as indicated by the BLRT, was supportive of the discriminant validity of PTSD and CPTSD. In this three-class solution, the largest class (64.5%) was characterised by high probabilities of meeting the diagnostic criteria for each of the PTSD and DSO symptom clusters; a symptom profile consistent with CPTSD. In contrast, a smaller (21.8%) class displayed a symptom profile whereby the probabilities of meeting the diagnostic criteria for the PTSD symptom clusters was high, and the probabilities of meeting the diagnostic criteria for the DSO symptom clusters was low; a symptom profile consistent with PTSD. Finally, a small class (13.6%) was characterised by low probabilities of meeting the diagnostic criteria for both PTSD and DSO clusters, with the exception of the Avoidance symptoms which were moderate. This symptom profile may reflect a third, more resilient group of refugees. Together, these findings add to a large and growing empirical literature supporting the discriminant validity of PTSD and CPTSD amongst multiple samples taken from culturally and trauma diverse backgrounds (Cloitre *et al.*, 2013, 2014; Elklit *et al.*, 2014; Knefel *et al.*, 2015; Perkonigg *et al.*, 2015; Murphy *et al.*, 2016; Karatzias *et al.*, 2017; Sachser *et al.*, 2017). Importantly, and in line with the ICD-11 principles of clinical utility and cross-cultural validity, this is the first study to provide support for the discriminant validity of PTSD and CPTSD amongst an (i) Arabic speaking; (ii) refugee sample; (iii) living in a low-resource, humanitarian setting.

The results of the multinomial logistic regression analysis found that only functional impairment significantly predicted class membership. This finding is consistent with previous studies (Cloitre *et al.*, 2013; Elklit *et al.*, 2014; Murphy *et al.*, 2016; Karatzias *et al.*, 2017), which indicate CPTSD class membership is associated with the highest levels of impairment and distress. Despite the fact that gender, unemployment status and type of traumatic exposure did not reach the level of statistical significance, the odds ratios for each of these variables were of a substantial magnitude and suggests the possibility of Type II errors. In previous studies, being female, unemployed and exposure to highly distressing traumatic events (e.g. Hyland *et al.*, 2017b) all demonstrated similar associations with PTSD and CPTSD class membership. The findings observed in the current study regarding the correlates of class membership are therefore largely consistent with those reported in previous studies.

Although the current study represents the first assessment of the discriminant validity of PTSD and CPTSD amongst a sample of refugees from the Middle East, there are a number of limitations that should be recognised. First, the small sample size limits the interpretability and generalisability of the findings. While a larger sample would have been preferable, practical constraints associated with accessing the sample limited the number of participants that could be recruited. Second, and relatedly, the time and resource demands associated with data collection limited the numbers of questions that could be asked of participants. Consequently, it was not possible to measure a number of important correlates of PTSD/CPTSD, such as the total number of (childhood and adulthood) traumatic life events, commonly co-occurring psychological distress factors (i.e. depression and psychosis), and risk and protective factors, including negative trauma-related cognitions and levels of social support. Third, as the current sample was comprised of treatment-seeking refugees, current results may overestimate the prevalence of PTSD and CPTSD amongst the wider (Syrian) refugee population.

In conclusion, the current results provide initial evidence of the discriminant validity of the ICD-11 proposals for PTSD and CPTSD amongst a sample of Arabic speaking Syrian refugees. While the limited sample size demands that these results not be over-interpreted, the similarity of our findings, when compared to those derived from multiple non-refugee, European clinical samples, suggests that the ICD-11 model of PTSD and CPTSD may well possess good cross-cultural validity. With an estimated 65.6 million people forcibly displaced globally, the potential reduction in the global burden of suffering via the application of an effective and efficient treatment of CPTSD symptoms amongst refugees is considerable. Indeed, our current results indicate that complex psychological responses to trauma are common amongst refugees. As standard best-practice treatments for PTSD (e.g. trauma-focused cognitive–behavioural therapy) tend to target fear-related symptoms, it may be that these treatments do not (optimally) treat the ‘Disturbances in Self-Organisation’ symptoms that characterise CPTSD. There is an emerging literature supporting the efficacy of phased-based interventions for CPTSD (e.g. ‘Skills Training in Affective and Interpersonal Regulation’: Cloitre *et al.*, 2002) which have been recognised within the Guidelines for the Treatment of CPTSD in adults, and developed by the International Society for Traumatic Stress Studies (Cloitre *et al.*, 2012). No studies have yet evaluated the efficacy of such treatments amongst trauma-exposed refugees. Given the psychological distress associated with CPTSD, and the vulnerable and unstable position that refugees often find themselves in, further research is necessary to determine how to most effectively and efficiently mitigate the trauma-related distress experienced by refugees.
